# Hydrophobic and Sticky Silver-Decorated Nanoimprinted ZnO Nanograss Substrates for Enhanced SERS Performance

**DOI:** 10.1021/acsami.5c07665

**Published:** 2025-06-26

**Authors:** Kuan-Ting Kuo, Wen-Huei Chang, Hsiang Chen, Jyun-Jie Chen, Chun-Hung Lin

**Affiliations:** † Department of Photonics, 34912 National Cheng Kung University, Tainan 70101, Taiwan; ‡ Department of Applied Chemistry, 63378 National Pingtung University, Pingtung 90003, Taiwan; § Department of Applied Materials and Optoelectronic Engineering, 59433 National Chi Nan University, Nantou 54561, Taiwan; ∥ Program on Key Materials, Academy of Innovative Semiconductor and Sustainable Manufacturing, National Cheng Kung University, Tainan 70101, Taiwan; ⊥ Meta-nanoPhotonics Center, National Cheng Kung University, Tainan 70101, Taiwan

**Keywords:** ZnO nanograss, hydrothermal method, sticky hydrophobic surface, surface-enhanced Raman spectroscopy, silver nanoparticles, nanoimprint lithography, selective growth, malachite green

## Abstract

This study successfully fabricated silver-decorated, submicrometer patterned zinc oxide (ZnO) nanograss substrates using nanoimprint lithography (NIL) and hydrothermal synthesis to achieve enhanced surface-enhanced Raman scattering (SERS) sensitivity. The ZnO nanograss structures were precisely patterned via NIL, allowing for controlled spatial arrangement and selective growth, with grating periods ranging from 1000 to 2000 nm and defined area widths between 500 and 1000 nm. Silver nanoparticles were deposited on the substrates through electron beam evaporation. The patterned design of the ZnO nanograss substrates significantly enhanced grating-mediated resonant excitation of localized surface plasmon resonance (LSPR), optimizing the interaction between incident light and the substrate. This resulted in more concentrated and focused light fields, which further amplified the LSPR effects. The impact of substrate hydrophobic characteristics, induced by dark storage for up to 3 months, on SERS performance was thoroughly investigated, with contact angles increasing from 93.5 to 144° during storage. These sticky properties facilitated the concentration of analyte molecules, significantly enhancing Raman signal intensity. Various periodic patterns, including one-dimensional (1D) gratings and two-dimensional (2D) arrays, were optimized to determine the ideal grating period for maximum Raman signal enhancement, achieving an analytical enhancement factor of 6.31 × 10^10^. Comprehensive characterization techniques, such as scanning electron microscopy (SEM), energy dispersive spectroscopy (EDS), X-ray diffraction (XRD), and X-ray photoelectron spectroscopy (XPS), were used to analyze the substrates’ morphology, elemental composition, and structural properties. SERS sensitivity was evaluated using malachite green (MG) molecules, revealing an impressive limit of detection (LOD) of 1.85 × 10^–15^. Furthermore, the substrates exhibited excellent long-term stability and signal reproducibility, maintaining consistent SERS performance after extended storage. This research establishes a cost-effective and highly sensitive SERS platform, offering significant potential for applications in chemical, environmental, and biochemical analysis.

## Graphical abstract

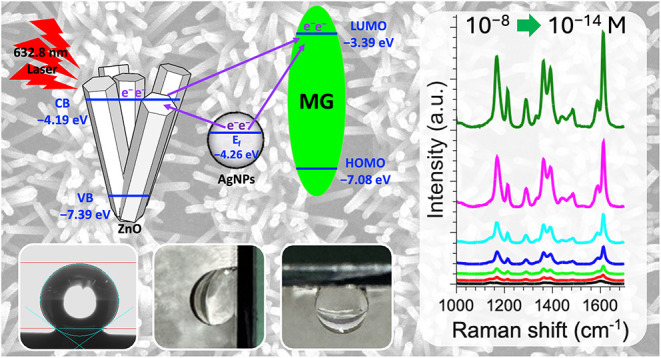

## 1 Introduction

Zinc oxide (ZnO) nanorods play a significant role in optoelectronic applications due to their unique physical, chemical, and optical properties. Their wide bandgap (approximately 3.37 eV) and high exciton binding energy (around 60 meV) make them highly suitable for ultraviolet photodetectors, enabling efficient light absorption and enhanced photoelectric conversion.^1,2^ Furthermore, ZnO nanorods grown in a patterned arrangement enable broadband photoresponses across the UV and visible spectrum, broadening their optoelectronic applications.^3^ In solar cells, ZnO nanorods serve electron transport layers, where their high surface area and nanostructured arrangement improve light harvesting and electron transport, boosting overall efficiency.^4,5^ Additionally, ZnO nanorods are widely used in light-emitting diodes (LEDs), particularly for UV or blue light emission, where their excellent crystalline quality ensures high photon emission efficiency.^6,7^ Their photocatalytic properties allow effective absorption of UV light to generate electron–hole pairs, enabling applications in environmental purification and water splitting for hydrogen production.^8,9^ Moreover, ZnO nanorods exhibit piezoelectric properties, making them ideal for piezoelectric and optoelectronic sensors that convert mechanical energy into electrical energy while enhancing sensing performance.^10^ In bio-optoelectronics, their chemical stability and biocompatibility enable applications in biosensors and optical bioimaging.^11,12^ Furthermore, ZnO nanorods are utilized in laser devices for room-temperature exciton lasers^13^ and as transparent conductive layers in touch panels and displays,^14,15^ where their nanostructure enhances conductivity and light transmittance. Overall, ZnO nanorods are a versatile material with broad applications in optoelectronics, and their integration with other materials and innovative fabrication methods continues to expand their potential in cutting-edge technologies.

ZnO nanorods have also emerged as promising substrates for surface-enhanced Raman scattering (SERS) due to their unique physical and chemical properties. Their high surface area and vertically aligned nanostructures facilitate effective adsorption of target molecules, enhancing molecule–substrate interactions to amplify Raman signals. Charge transfer enhancement is considered the dominant chemical enhancement mechanism, where photogenerated electrons or holes transfer directly to adsorbed molecules, altering their electronic structure and significantly enhancing their Raman scattering cross-section.^16–24^ This effect is particularly pronounced when ZnO nanorods are integrated with metallic nanoparticles such as silver or gold, where localized surface plasmon resonance (LSPR) facilitates additional charge transfer and provides strong electromagnetic enhancement. The synergistic combination of LSPR-driven electromagnetic enhancement and charge transfer enhancement results in a substantial increase in Raman signal intensity, making ZnO nanorods highly effective substrates for sensitive SERS detection.

Building on these foundational properties, recent advancements have leveraged ZnO’s structural versatility to expand its potential in SERS applications. Innovative designs such as jellyfish-like ZnO@Ag substrates,^19^ three-dimensional (3D) nanorod-grafted nanowire forests,^25^ hierarchically porous coralloid ZnO@Ag microspheres,^22^ silver-decorated ZnO nanoflowers,^23^ and surface-buckling-enhanced 3D metal/semiconductor devices^26^ have created abundant hot spots and facilitate charge transfer, significantly enhancing SERS performance. These substrates enable sensitive detection of analytes like melamine, malachite green, and thiram, while offering additional benefits such as recyclability, photocatalytic self-cleaning, and cost-effectiveness.

Traditional approaches to utilizing ZnO nanostructures as SERS substrates typically involve randomly distributed or disordered growth methods. These include chemically synthesized ZnO nanosphere powders and vertically aligned nanorod structures grown on flat substrates.^17,19,22–24,27–32^ These substrates are relatively simple to fabricate and provide a sufficient specific surface area, which helps facilitate the formation of hot spots and creates spaces for analyte molecules to be positioned near these hot spots, thereby enhancing the SERS signal.

Furthermore, ZnO nanorods grown in patterned arrangements using techniques like photolithography,^33^ periodic templates,^34^ and hierarchical 3D architectures^35,36^ enable precise structural control and induce enhanced light-trapping effects. These advancements have led to exceptional sensitivity and low detection limits. Advances in surface modification, morphology control, and hybrid material integration continue to optimize the structural design of ZnO-based SERS substrates, paving the way for versatile and efficient sensing technologies in food safety, environmental monitoring, and biochemical analysis.

In recent years, many studies have demonstrated that nanograss structures, owing to their high aspect ratio and surface roughness, are highly attractive for applications in optoelectronic sensing. For example, silicon nanograss combined with nanorod architectures has been shown to significantly reduce surface reflection, thereby improving light absorption efficiency in solar cells.^37^ Titanium dioxide nanograss, typically fabricated via anodic oxidation, exhibits strong electrochemical activity and is widely applied in photoelectrochemical and energy storage devices.^38^ In the field of SERS, gold nanograss structures synthesized using seed-mediated growth generate a high density of plasmonic hotspots. These hotspots intensify the local electromagnetic field, enabling highly sensitive detection of trace-level analytes.^39^ These studies collectively demonstrate the high potential of nanograss structures in enhancing specific surface area, strengthening light-matter interactions, and improving molecular capture efficiency.

Building on these insights and our previous work,^3^ where the nanoimprinted ZnO nanograss substrate demonstrated excellent broadband photodetection across the UV and visible spectrum, the present study extends the functionality of this platform to surface-enhanced Raman scattering (SERS) applications. The substrate was fabricated by combining nanoimprint lithography (NIL) and hydrothermal synthesis, followed by silver nanoparticle (AgNP) decoration via electron beam evaporation. NIL patterning addresses a key limitation of conventional ZnO nanorod-based SERS substrates, which often suffer from limited control over rod alignment and spatial distribution. By confining ZnO growth to predefined regions, the process enables the formation of well-organized blades of nanograss with improved structural uniformity and spatial precision. This ordered architecture facilitates more efficient plasmonic coupling and enhances interaction with the incident electromagnetic field. The subsequent AgNP modification leverages LSPR effects to further amplify the Raman signal, enabling highly sensitive analyte detection. This strategic transition from broadband photodetection to SERS detection highlights the versatility and adaptability of our ZnO nanograss platform, showcasing its potential for diverse optoelectronic and sensing applications.

Comprehensive characterization techniques, including scanning electron microscopy (SEM), energy-dispersive X-ray spectroscopy (EDS), X-ray diffraction (XRD), and X-ray photoelectron spectroscopy (XPS), were employed to analyze the morphology, elemental distribution, and structural properties of the substrates. Additionally, we investigated the influence of substrate hydrophobicity, achieved through prolonged dark storage, on SERS performance. Notably, the substrate exhibited strong adhesive properties that facilitated the concentration of analytes at specific locations, thereby enhancing SERS measurements.

This study highlights the advantages of the patterned design of ZnO nanograss substrates in LSPR coupling. The structural design was optimized by evaluating various periodic patterns, including 1D gratings and 2D arrays, to determine the optimal grating period for maximum signal amplification. SERS performance was assessed using malachite green (MG) molecules at ultralow concentrations to evaluate the limit of detection (LOD), sensitivity, and enhancement factor, and the results were compared with other ZnO-based SERS substrates reported in recent studies. Additionally, the long-term stability and reproducibility of the substrates were examined to ensure consistent SERS enhancement after extended storage. Ultimately, this research established a cost-effective and highly sensitive SERS platform for possible applications in chemical, environmental, and biochemical analysis.

## 2 Materials and Methods

### 2.1 Materials

Perfluoropolyether (PFPE)-urethane dimethacrylate (Fluorolink MD700) was purchased from Solvay Specialty Polymers (Bollate, Italy). 1,1,2-Trichloro-1,2,2-trifluoroethane was obtained from Grand Chemical Co. (Miaoli, Taiwan). 1*H*,1*H*,2*H*,2*H*-perfluorodecyltrichlorosilane (F13-TCS) was sourced from Alfa Aesar (Ward Hill, MA). Poly­(methyl methacrylate) (PMMA ACRYREX CM-211) was supplied by CHIMEI (Tainan, Taiwan). Zinc acetate dihydrate was acquired from Thermo Scientific (Reagent Lane, Fair Lawn). Hexamethylenetetramine and zinc nitrate hexahydrate were sourced from Thermo Scientific (Ward Hill, MA). Ag slug was purchased from ThinTech Materials Technology (Kaohsiung, Taiwan). MG was obtained from Sigma-Aldrich (St. Louis, MO).

### 2.2 Preparation of AgNP-Decorated Periodic ZnO Nanograss

Silicon (Si) substrates were cut into 1.25 cm × 1.25 cm pieces and cleaned using a piranha solution (H_2_SO_4_/H_2_O_2_, 3:1) for 30 min to remove organic contaminants, followed by sequential sonication in deionized (DI) water (10 min) and isopropanol (IPA, 5 min), then dried with nitrogen gas. A zinc acetate (Zn­(CH_3_COO)_2_) solution was spin-coated onto the Si substrates as a ZnO seed layer at 500 rpm for 5 s and 3000 rpm for 30 s, followed by heating at 120 °C for 5 min. This process was repeated five times to ensure uniform coverage. Next, a 5% PMMA solution was spin-coated at 3000 rpm for 30 s and soft-baked at 160 °C for 20 min. A PFPE mold was fabricated by drop-casting liquid PFPE onto a nanostructured Si master mold, followed by 365 nm UV curing.^40,41^ In the NIL process, the PFPE mold was pressed onto the PMMA-coated substrates, with a polyethylene terephthalate (PET) thin film placed on top to ensure uniform pressure distribution.^42^ Imprinting was carried out at 130 °C under 3 bar pressure for 10 min, after which the PFPE mold was removed using a precision blade. Oxygen plasma etching was then applied to remove the residual PMMA layer in nonpatterned regions, exposing the ZnO seed layer. ZnO nanograss was selectively grown via hydrothermal synthesis by immersing the substrates in a 0.05 M zinc nitrate hexahydrate (Zn­(NO_3_)_2_·6H_2_O) and 0.07 M hexamethylenetetramine (HMT) solution at 90 °C for 1.5 h. After growth, acetone was used to dissolve the PMMA resist, revealing the NIL-defined ZnO nanograss structure. To further enhance the SERS effect, the ZnO nanograss substrates were stored in a dark environment for 3 months, allowing the reduction of surface hydroxyl groups (OH^–^) and increasing hydrophobicity. Finally, 30 nm AgNPs were deposited onto the ZnO nanograss surface via electron beam evaporation, completing the fabrication of the SERS substrate.

### 2.3 SERS Spectrum Measurement

A 10 μL sample of the analyte, at varying concentrations, was applied to a SERS substrate and allowed to dry. SERS measurements were then performed using a modular Raman system (MRS-iHR320, HORIBA, Kyoto, Japan), equipped with a 632.8 nm He–Ne laser, and an Olympus BX53 optical microscope (Tokyo, Japan) with a 40× objective lens (numerical aperture, NA = 0.75). Baseline correction was applied using the built-in function of the LabSpec 5 spectroscopy software.

### 2.4 Characterization

SEM images and EDS were acquired using a field emission scanning electron microscope (SU8000, Hitachi, Japan) equipped with an EDS system. XRD data were obtained using a Multipurpose High-Intensity X-ray Thin-Film Micro Area Diffractometer (D8 DISCOVER Plus, Bruker). XPS analysis was performed using a PHI VersaProbe 4 system (Physical Electronics, Japan).

## 3 Results and Discussion

This study integrates NIL with the hydrothermal method to rapidly and efficiently fabricate ZnO nanograss structures arranged in a patterned configuration. NIL enables precise spatial control by defining specific growth regions on the substrate, thereby facilitating site-selective ZnO nanograss formation. The areas masked by PMMA patterns serve as growth-inhibited zones for ZnO. The hydrothermal method allows for straightforward tuning of nanorod dimensions during growth, enhancing the structural uniformity and functional performance of the resulting substrate. These features make the fabricated structures highly suitable for SERS applications. The fabrication process began with NIL patterning on a zinc acetate seed layer to define the designated growth areas. ZnO nanograss was then selectively grown within these predefined regions via the hydrothermal method. Finally, silver was deposited onto the ZnO nanograss surface to further boost SERS sensitivity. Figure 1 illustrates a schematic overview of the fabrication process for the AgNP-decorated, site-selective ZnO nanograss substrate.

**Figure 1:**
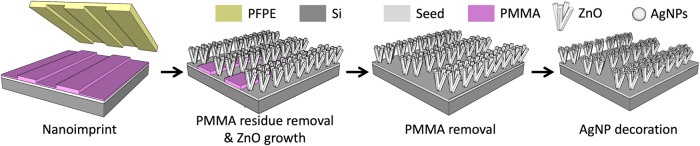
Schematic illustration of the fabrication process for the AgNP-decorated, site-selective ZnO nanograss substrate.

### 3.1 Surface Morphology and AgNP Decoration of ZnO Nanograss Substrates

The surface morphologies of the substrates were characterized by SEM, as shown in Figure 2. Figure 2a,b present the top-view and cross-sectional SEM images of the nonpatterned ZnO nanograss, respectively. The nanograss exhibited a randomly oriented and irregular growth pattern, yet it was uniformly distributed across the entire substrate surface. The average width of each nanorod was approximately 60 nm, with a maximum height of around 825 nm. This homogeneous coverage, combined with the high density and disordered alignment of the nanorods, significantly increased the specific surface area, which was favorable for the subsequent uniform decoration of AgNPs. Figure 2c,d illustrate the ZnO nanograss selectively grown in regions defined by a 1000 nm periodic 1D grating pattern, with the defining line width of the pattern being approximately 500 nm. Both top and cross-sectional views confirmed that the nanograss grew uniformly into the predefined periodic arrangements. In contrast to the predominantly vertical growth observed in the nonpatterned nanograss, the patterned nanograss, with ample space at the edges, allowed for lateral growth. This resulted in the ZnO nanorods growing at a slight tilt, giving the structure a more expansive, grass-like appearance. Figure 2e,f show the ZnO nanograss selectively grown in regions defined by 2D dot and hole arrays, respectively. The period and size of the defining areas were 1000 and 500 nm. The dot array structure clearly showed the growth of individual nanograss blades in a 2D periodic arrangement, while the hole array structure prevented the growth of nanograss in these areas. Figure 2g,h present images of the ZnO nanograss substrate after the deposition of a 30 nm thick Ag film. This deposition condition allowed Ag to form AgNPs on the ZnO nanograss. Both top-view and cross-sectional images revealed that the uniform distribution, yet disordered alignment, of the nanorods promoted the formation of AgNPs, which were evenly distributed on the surface and adhered firmly to the nanograss. The size of the AgNPs ranged from 10 to 50 nm. The AgNPs penetrated into the nanograss structure, with individual particles also visible along the sidewalls of the nanorods. The uniform distribution of AgNPs ensured consistent hot spot formation across the SERS substrate, leading to a more uniform and reliable enhancement of the SERS signal. The optical image of a 1D grating-patterned ZnO nanograss substrate is presented at the inset of Figure 2h, highlighting the uniformity of the patterned nanograss across a 5 mm × 5 mm area.

**Figure 2:**
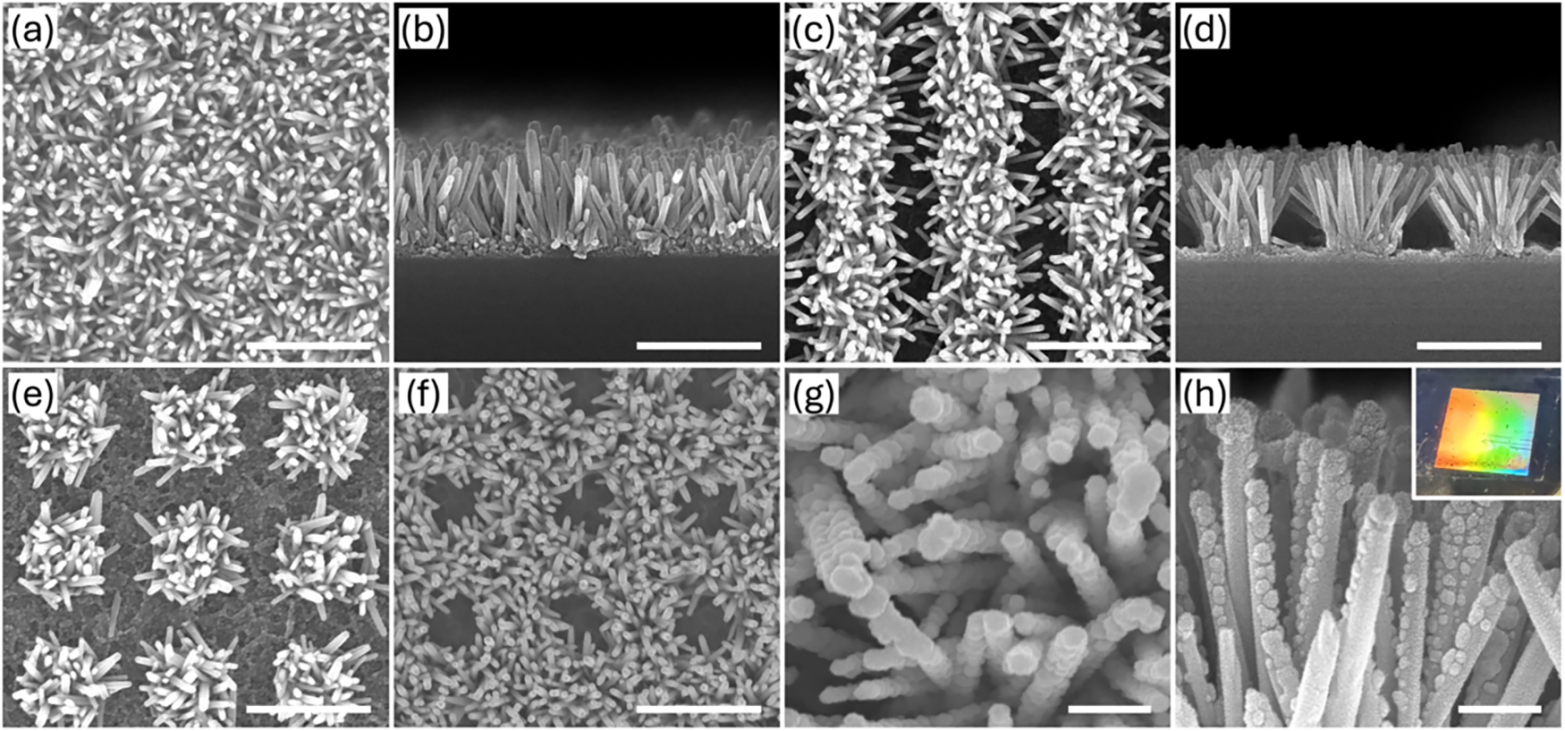
Morphology of ZnO nanograss. (a, b) SEM images of the nonpatterned ZnO nanograss, showing both top and cross-sectional views. (c, d) SEM images of ZnO nanograss selectively grown in regions defined by a 1000 nm periodic 1D grating pattern, with a defining line width of approximately 500 nm. (e, f) Top views of ZnO nanograss grown in regions defined by 2D dot and hole arrays, with a period of 1000 nm and line width of 500 nm. (g, h) Images of ZnO nanograss after deposition of a 30 nm thick Ag film, in both top and cross-sectional views. The optical image of the 1D grating-patterned ZnO nanograss substrate in the inset of (h) shows uniformity across a 5 mm × 5 mm area. The scale bars in (a–f) represent 1 μm, while those in (g, h) represent 200 nm.

### 3.2 EDS and XRD Analyses of AgNP-Decorated ZnO Nanograss Substrates

To further assess the elemental distribution on the patterned AgNP-decorated ZnO nanograss substrate, EDS was performed on the nanograss selectively grown in regions defined by a 1200 nm periodic 1D grating pattern, with the results shown in Figure 3. Figure 3a,f present top-view and cross-sectional SEM images of the patterned ZnO nanograss substrate, respectively. The corresponding EDS mapping results, illustrated in Figure 3b–e,3g–j, reveal the distribution of Zn, O, Si, and Ag. The EDS elemental analysis of the area depicted in the SEM images (Figure 3a,f) is further detailed in Figure S1 (Supporting Information, Note S1).

**Figure 3:**
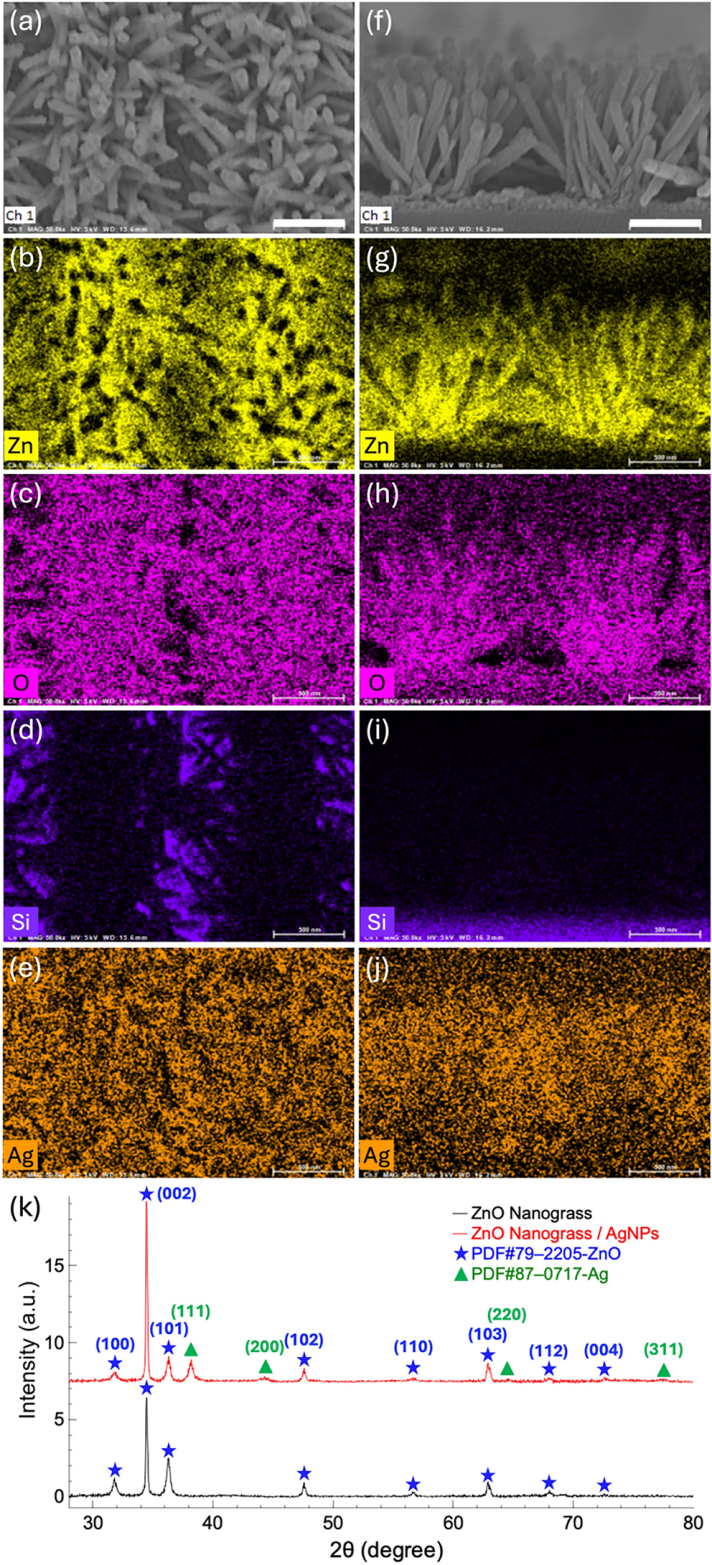
(a) Top-view SEM image and (f) cross-sectional SEM image of the ZnO nanograss substrate patterned with a 1200 nm periodic 1D grating. (b–e) EDS mapping of the elemental distribution of Zn, O, Si, and Ag for the top-view image in (a). (g–j) EDS mapping of the elemental distribution of Zn, O, Si, and Ag for the cross-sectional image in (f). (k) XRD analysis of the structural characteristics of the ZnO nanograss and the effects of AgNP modification. The scale bars in (a) and (f) represent 500 nm.

The Zn and O signals exhibited high-intensity regions corresponding to the nanograss growth areas arranged periodically, indicating uniform growth within these predefined regions. In contrast, the Si signal (Figure 3d) was predominantly observed in complementary regions where nanograss growth was inhibited, demonstrating that these areas were not covered by ZnO nanograss. This complementary periodic distribution validates the selective growth mechanism and highlights that the hydrothermal growth of ZnO was effectively confined to the predefined areas patterned by NIL. This confirms the precise patterning control of ZnO nanostructures achieved through the NIL technique.

Additionally, the Ag EDS mapping, shown in Figure 3e,j, reveals that Ag was uniformly distributed across the ZnO nanograss surface both laterally and vertically, with no evidence of aggregation. This confirms that electron beam evaporation resulted in consistent AgNP deposition. Such even distribution of AgNPs is critical for achieving consistent SERS enhancement, ensuring reliable and predictable amplification of the Raman signal.

XRD was used to further examine the structural characteristics of the ZnO nanograss and the impact of AgNP modification, as shown in Figure 3k. The XRD data reveal distinct diffraction peaks for the nonpatterned ZnO nanograss substrate at 2θ = 31.7, 34.4, 36.2, 47.5, 56.6, 62.9, 67.9, and 72.6°. These peaks correspond to the (100), (002), (101), (102), (110), (103), (112), and (004) crystal planes of the hexagonal wurtzite ZnO structure, according to the database (PDF card #79–2205).^35,43^ Notably, the (002) plane at 34.4° exhibits the strongest diffraction intensity, indicating that the ZnO nanograss preferentially grows along the *c*-axis, a characteristic typically observed in ZnO nanostructures. The distinct ZnO diffraction peaks, with no additional peaks from secondary phases, confirm the high crystallinity and purity of the sample.

After modification with AgNPs, the XRD spectrum of the ZnO substrate shows additional diffraction peaks at 2θ = 38.1, 44.3, 64.5, and 77.4°, corresponding to the metallic Ag peaks in the standard database (PDF card #87–0717).^44^ These peaks match the (111), (200), (220), and (311) crystal planes of metallic silver (Ag) with a face-centered cubic (FCC) structure. The peak at 38.1° for the (111) plane is the most prominent, demonstrating the high crystallinity of the AgNPs. The presence of these Ag diffraction peaks confirms the successful modification of AgNPs on the ZnO nanograss surface and the formation of metallic Ag, rather than its oxide forms (e.g., Ag_2_O or AgO).

Importantly, the XRD spectrum shows only peaks from ZnO and metallic Ag, with no additional peaks from impurities, confirming the high chemical purity of the sample. Furthermore, the modification with AgNPs does not affect the crystal structure of ZnO, which retains its stable hexagonal wurtzite form, suggesting that the AgNPs are simply attached to the surface of the ZnO nanograss without interacting with the ZnO lattice.

### 3.3 Effect of Storage Time on Hydrophobicity and SERS Performance of AgNP-Decorated ZnO Nanograss Substrates

We investigated the wettability of ZnO nanograss and its effect on SERS performance, focusing on the impact of prolonged storage. ZnO’s light-switchable wettability enables its surface to transition between hydrophilic and hydrophobic states.^45–47^ Hydrophobic surfaces are beneficial for SERS measurements, as they concentrate analytes and enhance signal sensitivity.^48–54^ In this study, we leveraged the hydrophobic and sticky properties of ZnO nanograss substrates to improve SERS performance. Using an AgNP-decorated ZnO nanograss substrate featuring a 1200 nm periodic 1D grating pattern, we evaluated the SERS performance of MG molecules at a concentration of 10^–8^ M. The study examined how the duration of dark storage impacts both the wettability of the ZnO surface and its SERS enhancement. Following dark storage, AgNPs were deposited onto the ZnO nanograss prior to conducting contact angle and SERS measurements.

To verify changes in surface wettability after prolonged dark storage, contact angle measurements were conducted using a 10 μL droplet. The freshly grown ZnO nanograss substrates, following AgNP deposition, exhibited weak hydrophobicity with contact angles around 93.5° (Figure 4a), which was attributed to the surface roughness of the ZnO nanograss. In contrast, substrates stored in the dark for 3 months prior to AgNP deposition exhibited significantly elevated contact angles during subsequent measurements, with values reaching up to 144° (Figure 4b), indicating a marked increase in hydrophobicity. XPS analysis (Supporting Information, Note S2) confirmed this observation, revealing a reduction in surface-adsorbed oxygen species over time, leading to decreased surface energy and a transition to a more hydrophobic state.

**Figure 4:**
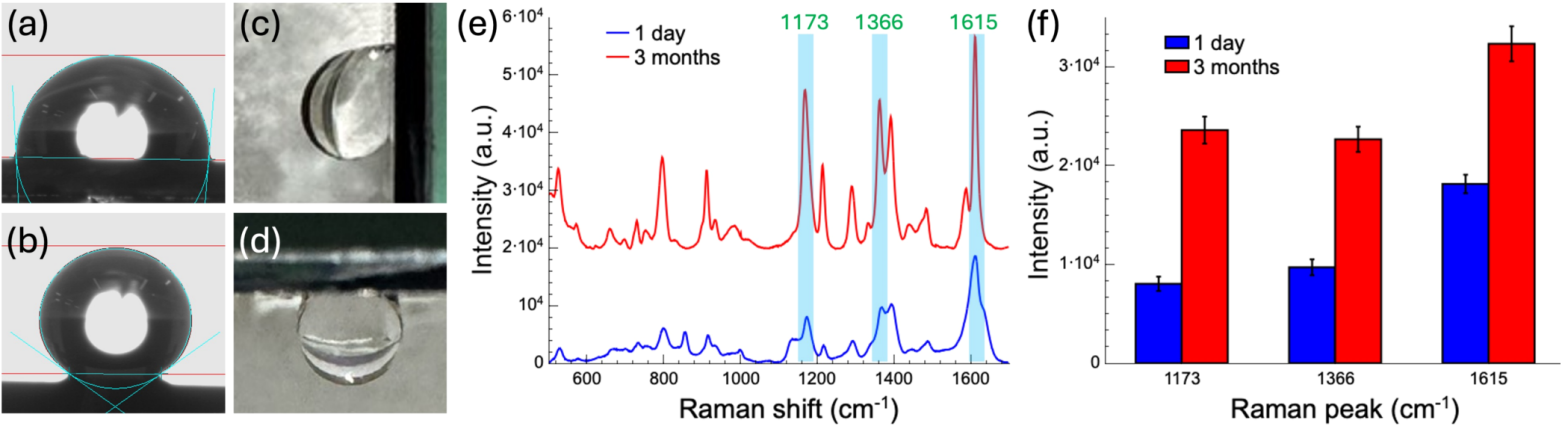
Wettability and SERS performance of AgNP-decorated ZnO nanograss substrates. (a) Contact angle of fresh substrates (∼93.5°). (b) Contact angle of substrates stored for three months (∼144°). (c, d) Images of a 10 μL water droplet on the three-month stored substrate at 90° (c) and 180° (d). (e) SERS spectra of MG molecules (10^–8^ M) on substrates stored for 1 day and three months, with average intensity from the five strongest signal points along the coffee ring. (f) Comparison of Raman peak intensities at 1173, 1366, and 1615 cm^–1^ for MG on substrates stored for 1 day and 3 months.

The transition in wettability of ZnO nanograss substrates plays a crucial role in their SERS performance. In the hydrophobic state, evaporating droplets form smaller coffee ring patterns, promoting greater aggregation of analyte molecules and enhancing local signal intensity. Furthermore, the surface exhibits sticky properties that aid in this process. Figure 4c,d display a 10 μL water droplet suspended on a substrate stored for 3 months, at tilt angles of 90 and 180°, respectively. The sticky hydrophobic surface facilitates the concentration of analytes at specific locations, providing a distinct advantage for SERS measurements.

Figure 4e compares the SERS spectra of MG molecules on AgNP-decorated ZnO nanograss substrates stored in a dark environment for 1 day and 3 months. The data reveal significant differences in SERS performance. The prominent peaks of MG appear at 1173, 1366, and 1615 cm^–1^, corresponding to the in-plane ring bending vibration of the C–H bond, symmetric stretching of the *N*-phenyl bond, and C–C stretching within the benzene ring, respectively.^50^ The intensities of these peaks are compared in Figure 4f. Freshly prepared substrates show moderate SERS enhancement, as their higher surface wettability causes the analyte solution to spread across the surface, resulting in lower Raman signal intensity. In contrast, substrates stored in the dark for 3 months exhibit increased hydrophobicity, which promotes smaller coffee ring patterns during droplet evaporation. This effect enhances local analyte concentration, leading to greater molecular aggregation and stronger Raman signals, thereby improving SERS sensitivity.

### 3.4 Enhancement of SERS Performance Using AgNP-Decorated ZnO Nanograss Substrates with Patterned Gratings

MG molecules at a concentration of 10^–7^ M were used as SERS probes to evaluate the enhancement performance of various substrates, including Ag thin films, Ag gratings, and both unpatterned and patterned AgNP-decorated ZnO nanograss substrates. All periodic patterns featured a 1D grating with a period of 1000 nm and a line width of 500 nm. The Ag layer on all samples was 30 nm thick, which was optimized for the patterned AgNP-decorated ZnO nanograss substrate (see Supporting Information, Note S3 and Figure S3). Figure 5a–b show the SERS spectra of MG, comparing the intensities of the three prominent Raman peaks at 1173, 1366, and 1615 cm^–1^ across the different substrates.

**Figure 5:**
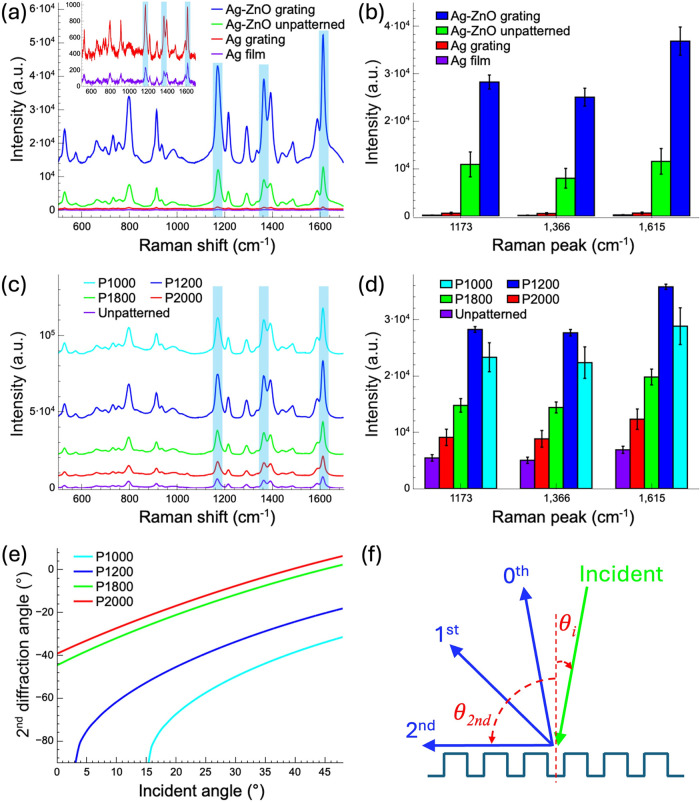
SERS performance on different substrates and grating periods. (a) SERS spectra of MG molecules (10^–7^ M) on Ag thin films, Ag gratings, and AgNP-decorated ZnO nanograss substrates with a 30 nm Ag layer. (b) Comparison of Raman peak intensities at 1173, 1366, and 1615 cm^–1^ for the substrates in (a). (c) SERS spectra of MG molecules (10^–8^ M) on ZnO nanograss substrates with different grating periods (1000, 1200, 1800, and 2000 nm). (d) Comparison of Raman peak intensities at 1173, 1366, and 1615 cm^–1^ for the substrates in (c). The SERS spectra were averaged from the five strongest points along the coffee ring for each sample. (e) Diffraction angles for second-order diffraction corresponding to incident angles ranging from 0° to 48.6° for four different grating periods. (f) Schematic of the grating structure, showing the incident angle of light and the corresponding diffraction angles for various diffraction orders.

For the 30 nm thick Ag film on a Si substrate, only weak Raman peaks of MG were observed, indicating minimal SERS enhancement. This is due to the smooth, featureless Ag film, which lacks the surface roughness or nanoscale structures needed to generate plasmonic hot spots. Without these hot spots, the LSPR effect is weak, resulting in limited Raman signal enhancement and poor SERS sensitivity. In contrast, the 30 nm thick Ag grating on a Si substrate showed moderate Raman signal enhancement, mainly due to the coupling of incident light to Surface Plasmon Polaritons (SPPs) at the grating. The periodic nature of the grating facilitates diffraction, efficiently coupling light into SPPs that propagate along the metal surface, amplifying the electromagnetic field and strengthening the SERS signal.

Further enhancement in the Raman signal was observed when nonpatterned ZnO nanograss substrates were used with the same 30 nm Ag coating. The Ag deposition formed AgNPs on the ZnO nanograss, as shown in Figure 2g,h. The MG Raman peaks became significantly stronger due to two key mechanisms. First, the vertically aligned ZnO nanograss offers a significantly larger surface area compared to flat substrates. Its increased surface area and roughness provide numerous sites for AgNP attachment, creating a favorable environment for the formation of densely packed AgNPs. This enhanced nanoparticle distribution increases the density of plasmonic hot spots, ultimately enhancing the SERS performance. Second, the ZnO–AgNP interface enhances the SERS response primarily through the LSPR of AgNPs, which generates strong electromagnetic fields under 632.8 nm laser excitation, amplifying the Raman signal. Hot electron transfer from AgNPs to the ZnO conduction band further modifies molecule–substrate interactions, boosting Raman activity.^21–24^ ZnO surface states and defects facilitate charge transfer between AgNPs and ZnO, contributing to the enhanced signal.^55^ Finally, plasmonic fields improve carrier dynamics by reducing electron–hole recombination, sustaining more active charge carriers.^56,57^

Finally, when the ZnO nanograss was grown in a patterned arrangement, including 1D grating and 2D dot and hole arrays, the SERS signal was significantly further amplified (see Figure 5a,b and Supporting Information, Note S4 and Figure S4). Among these, the 1D grating arrangement exhibited the most significant enhancement. The patterned ZnO substrate outperformed the nonpatterned substrate, as the periodic structures effectively enhanced the localized electromagnetic fields by coupling with incident light, increasing the density of plasmonic hot spots. Additionally, these highly ordered arrangements of ZnO nanograss significantly enhanced light trapping capabilities, allowing incident light to undergo multiple scattering and reflection within the nanograss, further boosting the SERS signal.^36^ This grating arrangement optimized the interaction between the analyte molecules and the enhanced electromagnetic fields, facilitating stronger molecular excitation and leading to more pronounced Raman scattering.

### 3.5 Effect of Grating Period on SERS Performance of ZnO Nanograss Substrates

Building on previous findings that ZnO nanograss substrates with grating patterns enhance Raman signals in SERS applications, we further investigate the effect of varying the grating period on SERS performance. To maintain a consistent structural ratio, all grating designs were fabricated with a fixed period-to-line width ratio of 2:1. A nonpatterned substrate and patterned substrates with periods of 1000, 1200, 1800, and 2000 nm were compared. SERS spectra of MG molecules at a concentration of 10^–8^ M were measured to identify the optimal grating configuration for maximum SERS enhancement, as shown in Figure 5c. Figure 5d compares the intensities of the three prominent Raman peaks across the different substrates.

Among the tested designs, the ZnO nanograss substrate with a 1200 nm period produced the strongest Raman signal. This enhancement is attributed to the optimal coupling between the grating structure and LSPR modes at this specific periodicity. The LSPR effect is likely because the ZnO substrate is decorated with discrete AgNPs rather than continuous Ag films, which would facilitate SPP excitation. The regularity and periodicity of the 1D grating enhance the interaction between incident light and the substrate, resulting in a more concentrated and focused light field. Given that the objective lens has an NA of 0.75, the corresponding incident angle for focusing on the SERS substrate ranges from 0 to 48.6°. Figure 5e shows the diffraction angles for second-order diffraction across four different grating periods, corresponding to incident angles between 0 and 48.6°. Figure 5f displays a schematic of the grating structure, illustrating the incident angle and the corresponding diffraction angles for various diffraction orders. At an incident angle of 3.1°, the 1200 nm grating results in a diffraction angle of 90°, which is parallel to the grating plane. This configuration achieves the most favorable resonance condition, allowing for efficient excitation of plasmonic modes and an enhanced local electromagnetic field, thereby intensifying the Raman scattering effect. The 1000 nm grating, at a 15.4° incident angle, also produces a diffraction angle of 90°, resulting in a moderate signal enhancement, although not as pronounced as with the 1200 nm design. As the grating period increased to 1800 and 2000 nm, a noticeable decline in Raman signal intensity was observed. This reduction is likely due to a mismatch between the larger periodic structures and the plasmonic resonance conditions, which diminishes LSPR efficiency and, consequently, SERS sensitivity. Based on these findings, the optimized 1200 nm grating configuration will be utilized in subsequent Raman measurements to ensure maximum sensitivity.

### 3.6 Sensitivity and LOD of AgNP-Decorated ZnO Nanograss Substrates

To further evaluate the sensitivity and LOD of the SERS substrate, measurements were conducted using ZnO nanograss substrates with a 1200 nm 1D grating. MG molecules at concentrations ranging from 10^–8^ to 10^–14^ M were tested to assess the substrate’s detection capability and signal intensity at ultralow concentrations.

Figure 6a shows a clear and consistent decrease in Raman signal intensity as the MG concentration is reduced from 10^–8^ to 10^–14^ M, highlighting the high sensitivity of the SERS substrate. The SERS spectra were averaged from the five highest-intensity signal points along the coffee ring for each sample. Even at an extremely low concentration of 10^–14^ M, the characteristic Raman peaks of MG at 1173, 1366, and 1615 cm^–1^ remain detectable. However, at 10^–15^ M, the Raman signals become indistinguishable due to interference from the substrate’s background noise. A strong linear correlation (*R* ^2^ = 0.993) between MG concentration and SERS intensity at 1615 cm^–1^, shown in Figure 6b, indicates excellent signal responsiveness and quantification capability across a wide concentration range. The linear regression equation is given by

1 log(SERSintensity)=B×log(analyteconcentration)+A

where *A* is the intercept and *B* is the slope of the regression line. This linearity reflects the substrate’s stable enhancement effect and high reproducibility, making it ideal for detecting trace-level concentrations of target molecules. Using the peak at 1615 cm^–1^ as a reference, the limit of detection (LOD) for MG was calculated to be 1.85 × 10^–15^ M. This was determined using the formula

2 $$\log\left(\mathrm{LOD}\right)=\frac{\log\left(I_{\mathrm{blank}}+3\sigma_{\mathrm{blank}}\right)-A}{B}$$

where *I*_blank_ represents the mean signal of the blank sample and σ_blank_ is the standard deviation of the blank signal. The observed linearity, along with the calculated LOD, highlights the potential of this substrate for sensitive detection of trace-level concentrations.

**Figure 6:**
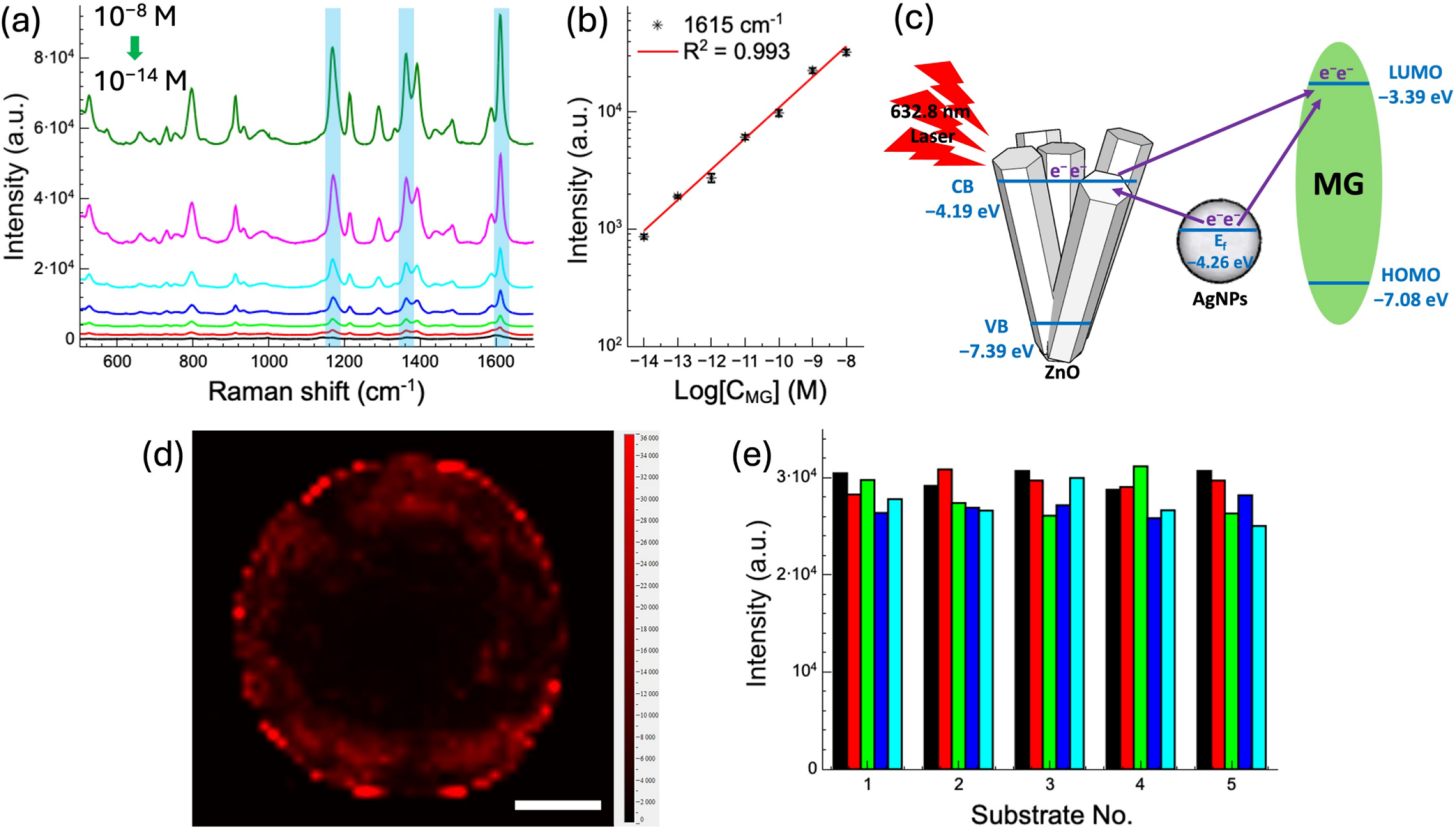
(a) SERS spectra of MG in DI water at concentrations from 10^–8^ to 10^–14^ M on ZnO nanograss substrates with a 1200 nm 1D grating. The SERS spectra were averaged from the five strongest points along the coffee ring for each sample. (b) Linear correlation (*R* ^2^ = 0.993) between MG concentration and SERS intensity at 1615 cm^–1^ in DI water. (c) Schematic illustration of the energy band alignment among ZnO, AgNPs, and MG molecules. (d) Raman mapping image at 1615 cm^–1^ with MG at 10^–8^ M. The scale bar represents 500 μm. (e) Reproducibility assessment of the SERS substrate using five prepared samples with 10^–8^ M MG, yielding an RSD of 6.45%.

The AgNP-decorated ZnO nanograss substrate, featuring a 1D grating pattern fabricated through NIL, exhibits remarkable SERS enhancement. Capable of detecting molecules as low as 10^–14^ M, this substrate emerges as a promising platform for ultrasensitive SERS detection in both chemical and biological applications. To further quantify the SERS enhancement achieved by this substrate, the analytical enhancement factor (AEF) was determined to be 6.31 × 10^10^ using the following equation^58^

3 $$\mathrm{AEF}=\frac{I_{\mathrm{SERS}}/C_{\mathrm{SERS}}}{I_{\mathrm{Raman}}/C_{\mathrm{Raman}}}$$

where *I*_SERS_ and *C*_SERS_ represent the SERS intensity (861.1) and MG concentration (10^–14^ M) measured on the ZnO nanograss substrate, while *I*_Raman_ and *C*_Raman_ represent the Raman intensity (1365.1) and MG concentration (10^–3^ M) obtained from a bare Si substrate. This value highlights the substrate’s exceptional enhancement capabilities, positioning it as a highly effective platform for ultrasensitive molecular detection.

The significant enhancement of Raman signals in the AgNP-decorated ZnO nanograss SERS substrate can be attributed to the synergistic effects of both electromagnetic and chemical enhancement mechanisms, with the charge transfer process playing a particularly critical role. A schematic illustration of the energy band alignment between ZnO, AgNPs, and MG molecules is shown in Figure 6c. Under 632.8 nm laser excitation, the AgNP generate LSPR effects, leading to the formation of high-intensity electromagnetic hotspots at the interparticle gaps. These hotspots induce the generation of numerous hot electrons, which can be directly injected from the Fermi level of Ag into the lowest unoccupied molecular orbital (LUMO) of the MG molecules adsorbed on the surface. This direct charge injection alters the electronic structure and polarizability of the MG molecules, thereby enhancing their Raman scattering cross-section.^24^ Additionally, the structural characteristics of the ZnO nanograss further facilitate the charge transfer process. Its high specific surface area provides numerous anchoring sites for AgNPs and ample surface area for analyte adsorption, creating abundant effective hotspots. Furthermore, the conduction band (CB) level of ZnO lies between the Fermi level of Ag and the LUMO level of the MG molecules, acting as an energy bridge that enables hot electrons to transfer across a smaller energy gap to the molecular orbitals of MG.^23^ In this ternary heterostructure (Ag–ZnO–MG), once LSPR is excited, electrons can first be injected from Ag into the CB of ZnO and subsequently transferred from ZnO to the LUMO of the MG molecules. This indirect yet energetically favorable charge transfer pathway further amplifies the SERS signal intensity.

The uniformity of the SERS substrates, both across the entire surface and within localized regions, was confirmed through optical imaging, morphological analysis, and elemental composition characterization, as shown in Figures 2 and 3. However, the SERS measurement approach leveraged the substrate’s hydrophobic properties, which caused MG molecules to accumulate at the coffee ring edge. This aggregation significantly enhanced SERS sensitivity but led to an uneven spatial distribution of signals, as shown in the Raman mapping image in Figure 6d. For this mapping, a 10 μL droplet of 10^–8^ M MG solution was deposited onto the substrate, and Raman measurements were taken at 50 μm intervals. The resulting intensity map of the 1615 cm^–1^ MG peak displayed a circular pattern, indicating that most molecules were concentrated along the coffee ring.

To assess the reproducibility of the SERS substrates, five independently prepared samples were tested using 10^–8^ M MG. For each sample, the five strongest signal points along the coffee ring at 1615 cm^–1^ were analyzed, as shown in Figure 6e. The results demonstrated consistent spectral responses across all samples, with a relative standard deviation (RSD) of 6.45% calculated from the mean intensity of each substrate, confirming the substrates’ reliability for SERS measurements.

Compared to recent ZnO-based SERS detection studies, this approach not only demonstrates exceptional sensitivity but also achieves an outstanding enhancement factor. Table 1 summarizes recent research on ZnO-based SERS detection, highlighting studies that either use the 632.8 nm laser wavelength for MG detection (potentially with different light sources) or employ the same wavelength for R6G detection.

**Table 1:** Summary of Recent ZnO-Based SERS Detection Studies, Including Those Utilizing the 632.8 nm Laser Wavelength for MG Detection (with Varying Light Sources) or for R6G Detection

SERS substrate/metal nanoparticles	laser wavelength (nm)	analyte	detection limit (M)	enhancement factor	enhancement factor definition^a^	refs
jellyfish-like ZnO@Ag hybrid structure	633	R6G	10^–11^	7.58 × 10^6^	EF	^19^
ZnO nanorod-grafted nanowire forests with Au nanoparticles	632.8	R6G	10^–10^	6.4 × 10^6^	AEF	^25^
coralloid ZnO@Ag microspheres	532	MG	10^–9^	1.61 × 10^7^	N.A.	^22^
ZnO nanoflowers decorated with Ag nanoparticles	633	MG	10^–9^	2.98 × 10^7^	N.A.	^23^
Ag nanoparticles decoration of flower-like ZnO nanorods on a buckled substrate	632.8	R6G	10^–9^	2.43 × 10^8^	AEF	^26^
hydrophobic and sticky nanoimprinted ZnO nanograss	632.8	MG	10^–14^	6.31 × 10^10^	AEF	this work

a AEF: refers to eq 3; EF is defined as $\mathrm{EF}=\frac{I_{\mathrm{SERS}}/N_{\mathrm{SERS}}}{I_{\mathrm{Raman}}/N_{\mathrm{Raman}}}$ , where *I*_SERS_ and *N*_SERS_ represent the SERS intensity and the number of analyte molecules in the scattering volume for the SERS measurement, while *I*_Raman_ and *C*_Raman_ represent the Raman intensity and the number of analyte molecules in the scattering volume for the Raman (non-SERS) measurement.^58^

### 3.7 Detection of MG in River Water

To evaluate the practical applicability of the SERS substrate developed in this study, MG was spiked into water samples collected from the Zhuxi Stream in downtown Tainan to simulate real-world detection conditions. The river water served was used as the solvent to prepare a series of MG solutions with concentrations ranging from 10^–8^ to 10^–14^ M. SERS measurements were then performed using the fabricated substrates, as shown in Figure 7a,b. As illustrated in Figure 7a, distinct Raman spectral features of MG remained clearly identifiable across the entire concentration range. Remarkably, the characteristic peaks of MG were still detectable even at the extremely low concentration of 10^–14^ M, indicating that the substrate maintains stable detection performance in complex aqueous environments. Figure 7b presents a quantitative analysis based on the peak intensity at 1615 cm^–1^, a characteristic Raman band of MG, which shows a strong linear correlation between concentration and signal intensity (*R* ^2^ = 0.985). Despite the presence of potential interferents such as impurities and suspended particles in the river water, the SERS substrate consistently produced clear and reproducible Raman signals. This robust quantification capability in spiked environmental samples highlights the substrate’s potential for future development of trace-level pollutant detection platforms.

**Figure 7:**
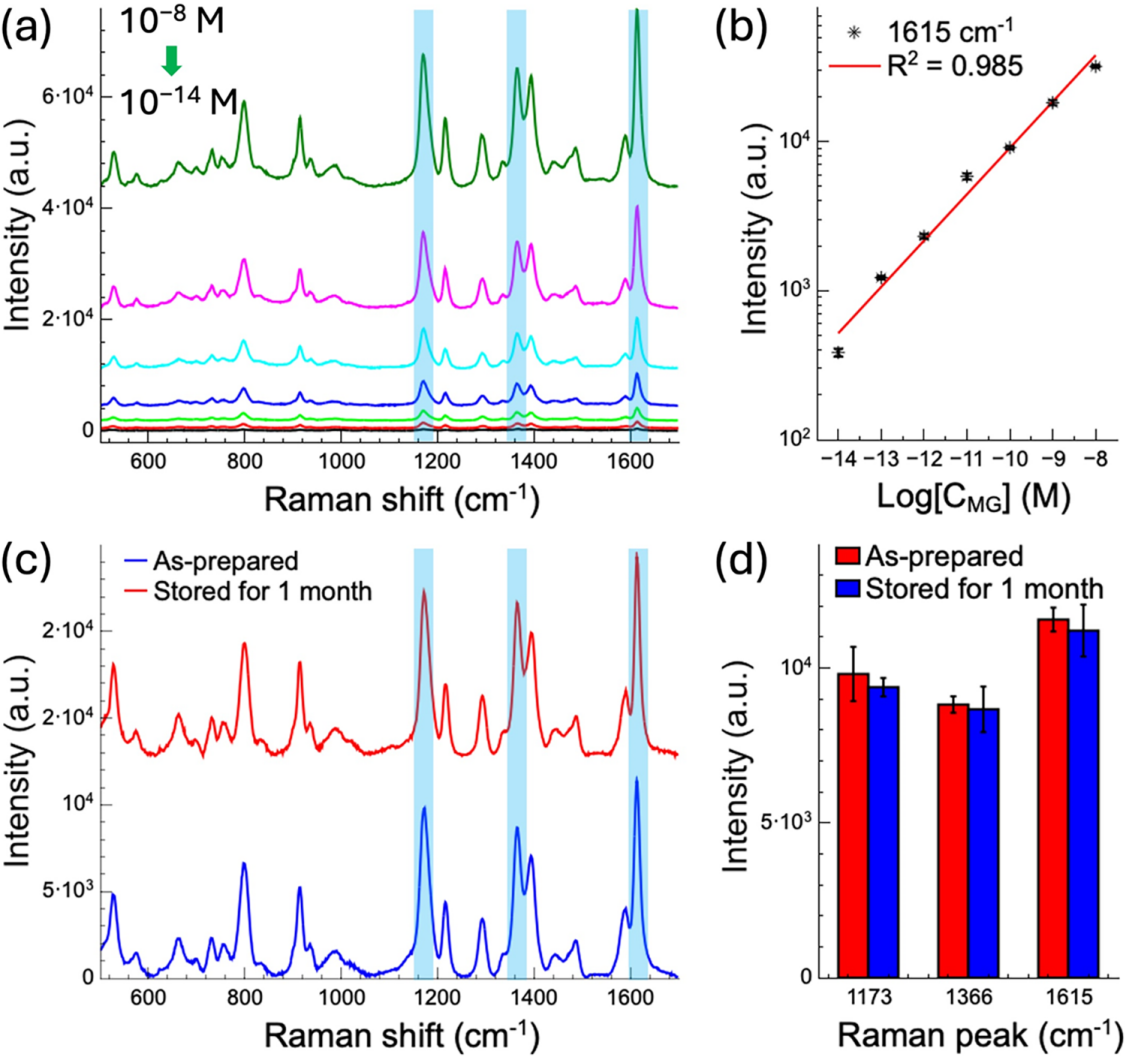
(a) SERS spectra of MG spiked in river water at concentrations from 10^–8^ to 10^–14^ M on ZnO nanograss substrates. (b) Linear correlation (*R* ^2^ = 0.985) between MG concentration and SERS intensity at 1615 cm^–1^ in river water. (c) SERS spectra of MG (10^–8^ M) obtained from a freshly prepared substrate and after one month of storage at room temperature. (d) Comparison of SERS intensities at 1173, 1366, and 1615 cm^–1^ across both time points. All SERS spectra were averaged from the five strongest points along the coffee ring for each sample.

### 3.8 Long-Term Stability of the SERS Substrates

Finally, we investigated the long-term stability of the patterned 1D grating AgNP-decorated ZnO nanograss substrate. Figure 7c presents a comparison between two SERS substrates one measured immediately after AgNP deposition and the other measured after being stored at room temperature for one month. For each measurement, a 10 μL solution of MG at a concentration of 10^–8^ M was applied to the substrate and allowed to dry prior to analysis. The freshly prepared SERS substrate demonstrated effective enhancement of the Raman signals of MG molecules, exhibiting clear and distinct characteristic peaks. Remarkably, after one month of storage, the second substrate displayed only minimal changes in signal intensity, indicating negligible degradation of the Raman response over time. Figure 7d illustrates the SERS intensity comparison of three prominent Raman peaks recorded at both time points.

The SERS substrates exhibit excellent long-term stability and high reproducibility, maintaining strong enhancement even after prolonged storage. This remarkable stability is attributed to two key factors. First, the ZnO nanograss substrate exhibits exceptional chemical stability due to its well-defined crystalline structure and robust chemical properties, which make it resistant to environmental degradation. Second, the optimized 30 nm Ag deposition effectively prevents AgNP aggregation, and the AgNPs are resistant to oxidation when stored at room temperature. This ensures stability for at least one month, preserves the LSPR effect, and guarantees long-lasting SERS signal enhancement.

## 4 Conclusions

This research successfully demonstrated the fabrication and optimization of AgNP-decorated, submicro patterned ZnO nanograss substrates using NIL and hydrothermal synthesis for enhanced SERS sensitivity. The ZnO nanograss structures were precisely patterned with grating periods ranging from 1000 to 2000 nm and defined area widths from 500 to 1000 nm, enabling controlled spatial arrangements. The patterned design of the ZnO nanograss substrates significantly enhanced LSPR through grating-coupled effects, optimizing the interaction between incident light and the substrate. This led to more concentrated and focused light fields, further amplifying the LSPR effects. The study also revealed that the hydrophobic characteristics of the substrate, induced by dark storage for up to three months, greatly enhanced SERS performance. The contact angle increased from 93.5 to 144°, and these hydrophobic properties significantly promoted the concentration of analyte molecules, leading to a substantial amplification of the Raman signal.

Through structural optimization, including the evaluation of periodic patterns and grating periods, the substrates achieved maximum Raman signal amplification, with an AEF of 6.31 × 10^10^. Comprehensive characterization confirmed the substrates’ high-quality morphology and structural integrity, while SERS testing with MG molecules demonstrated ultralow LOD down to 1.85 × 10^–15^ M. Additionally, the substrates exhibited excellent long-term stability and signal reproducibility, maintaining consistent SERS performance after extended storage.

From a fabrication perspective, NIL offers a low-cost and high-throughput approach for large-area patterning, with good structural fidelity that supports scalable nanostructure fabrication. In parallel, hydrothermal growth provides a simple, safe, and energy-efficient method for synthesizing vertically aligned ZnO nanostructures under mild processing conditions. This technique is compatible with a range of substrate types, including flexible materials, and has the potential for scale-up. The combination of NIL and hydrothermal synthesis allows for controlled nanoscale structural formation while maintaining reasonable manufacturing feasibility, suggesting a viable route toward cost-effective and adaptable sensing platforms.

In summary, this study presents a ZnO-based SERS substrate with promising sensitivity and structural tunability, developed through accessible and potentially scalable fabrication methods. While further work is needed to fully evaluate its performance across real-world conditions and broader sensing applications, the platform demonstrates encouraging potential for future development in chemical, environmental, and biochemical sensing contexts.

## Supplementary Material



## Notes

EDS analysis of AgNP-decorated ZnO nanograss substrate, XPS characterization of AgNP-decorated ZnO nanograss, optimization of Ag thickness on ZnO nanograss for SERS enhancement, and a comparison of SERS intensity on AgNP-decorated substrates with various patterns (PDF)
