# Supplementary material for: Hydrophobic and Sticky Silver-Decorated Nanoimprinted ZnO Nanograss Substrates for Enhanced SERS Performance
Source: ACS Appl Mater Interfaces. 2025 Jun 26;17(27):39448–60. doi: 10.1021/acsami.5c07665 (PMC12257462; doi:10.1021/acsami.5c07665)
Supplement: Supplementary file 1 [file am5c07665_si_001.pdf]

Supporting Information

Hydrophobic and Sticky Silver-Decorated Nanoimprinted ZnO Nanograss Substrates for Enhanced SERS Performance

Kuan-Ting Kuo [a], *Wen-Huei Chang* [b,*], *Hsiang Chen* [c], *Jyun-Jie Chen* [a], *Chun-Hung Lin* [a,d,e,*]

[a] Department of Photonics, National Cheng Kung University, Tainan 70101, Taiwan

[b] Department of Applied Chemistry, National Pingtung University, Pingtung 90003, Taiwan

[c] Department of Applied Materials and Optoelectronic Engineering, National Chi Nan University, Nantou 54561, Taiwan

[d] Program on Key Materials, Academy of Innovative Semiconductor and Sustainable Manufacturing, National Cheng Kung University, Tainan 70101, Taiwan

[e] Meta-nanoPhotonics Center, National Cheng Kung University, Tainan 70101, Taiwan

*Correspondence: Wen-Huei Chang, Department of Applied Chemistry, National Pingtung University, No.1, Linsen Rd., Pingtung 90003, Taiwan, E-mail: whchang@mail.nptu.edu.tw; Chun-Hung Lin, Department of Photonics, National Cheng Kung University, No.1, University Rd., Tainan 70101, Taiwan, E-mail: chlin@ncku.edu.tw

Note S1. EDS Analysis of AgNP-decorated ZnO nanograss substrate

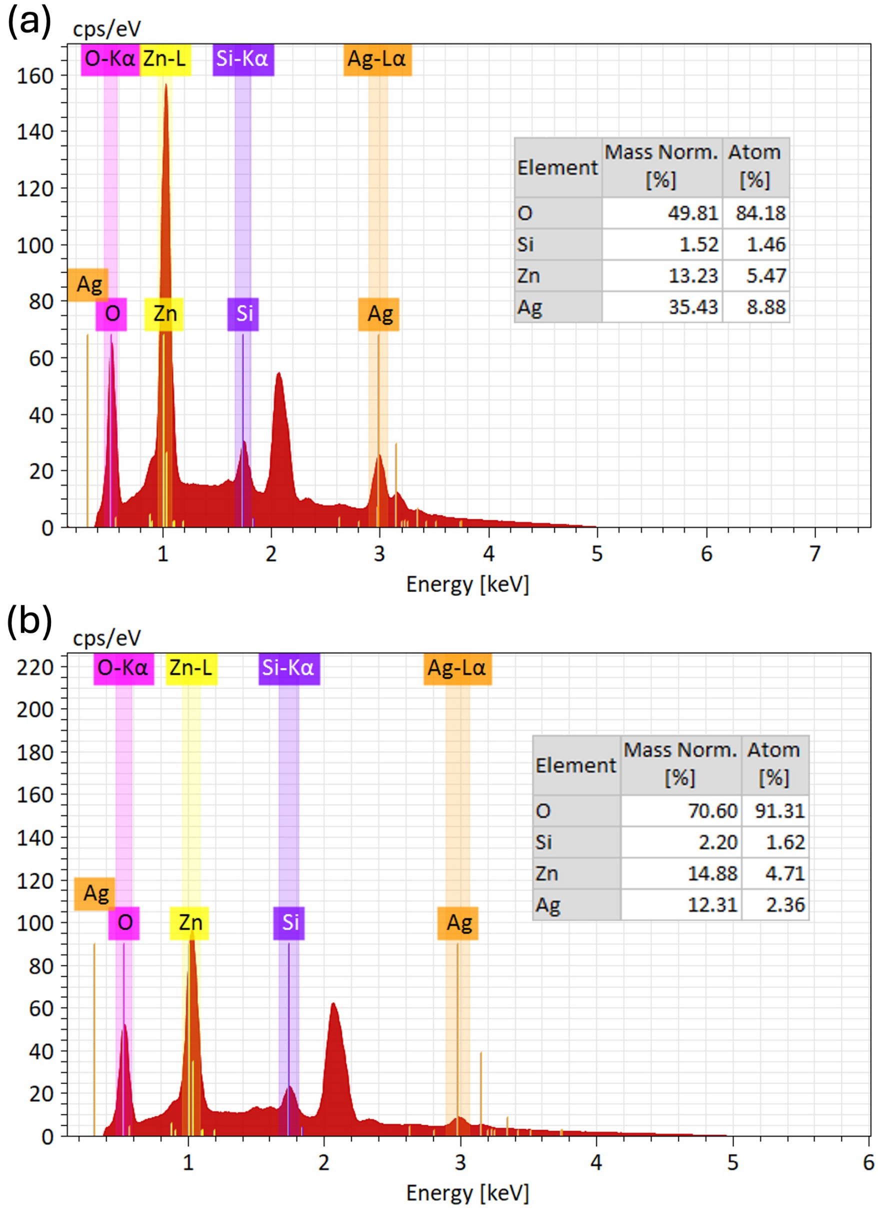

Figure S1. The EDS elemental analysis of the area depicted in the SEM images shown in Figure 3a and Figure 3f.

Note S2. XPS Characterization of AgNP-Decorated ZnO Nanograss

Figure S2 shows the X-ray Photoelectron Spectroscopy (XPS) analysis of the bonding states of Zn, Ag, and O elements on the surface of AgNP-decorated ZnO nanograss substrates. Panel S2a presents the XPS data for the sample stored in a dark environment for one day, while panel S2b shows the data for the sample stored for three months. AgNPs were deposited onto both samples before XPS analysis.

The Zn 2p spectra for both samples exhibit two main peaks at 1021.5 eV (Zn $2p_3/_2$) and 1044.6 eV (Zn $2p_1/_2$), corresponding to the characteristic peaks of Zn^{2+} in ZnO, indicating that Zn predominantly exists as ZnO.[1, 2] These peaks align with standard binding energy values for ZnO reported in the literature. The Ag 3d spectra reveal the bonding state of AgNPs, with main peaks observed at 367.5 eV (Ag $3d_5/_2$) and 373.5 eV (Ag $3d_3/_2$), indicating that Ag predominantly exists in its metallic state (Ag^0) on the surface.[3] These peaks match the standard binding energies of metallic silver, with no peaks corresponding to silver oxides (Ag_2O or AgO), confirming that the AgNPs deposited by evaporation did not undergo oxidation.

The O 1s spectrum shows two main peaks at 530.5 eV and 531.9 eV. The peak at 530.5 eV indicates oxygen in the ZnO lattice, reflecting the integrity of the ZnO structure, while the peak at 531.9 eV is associated with surface-adsorbed oxygen species, such as hydroxyl groups (OH^-), oxygen vacancies, or other active oxygen species commonly found on metal oxide surfaces.[1, 4] After three months of storage, the peak at 531.9 eV decreases significantly, suggesting a reduction in surface-adsorbed oxygen species. This reduction is likely due to the desorption or transformation of hydroxyl groups, leading to increased hydrophobicity of the AgNP-decorated ZnO nanograss substrates.[5, 6] This trend, where a decrease in surface oxygen species enhances hydrophobicity, has been observed in other ZnO materials as well.[7, 8]

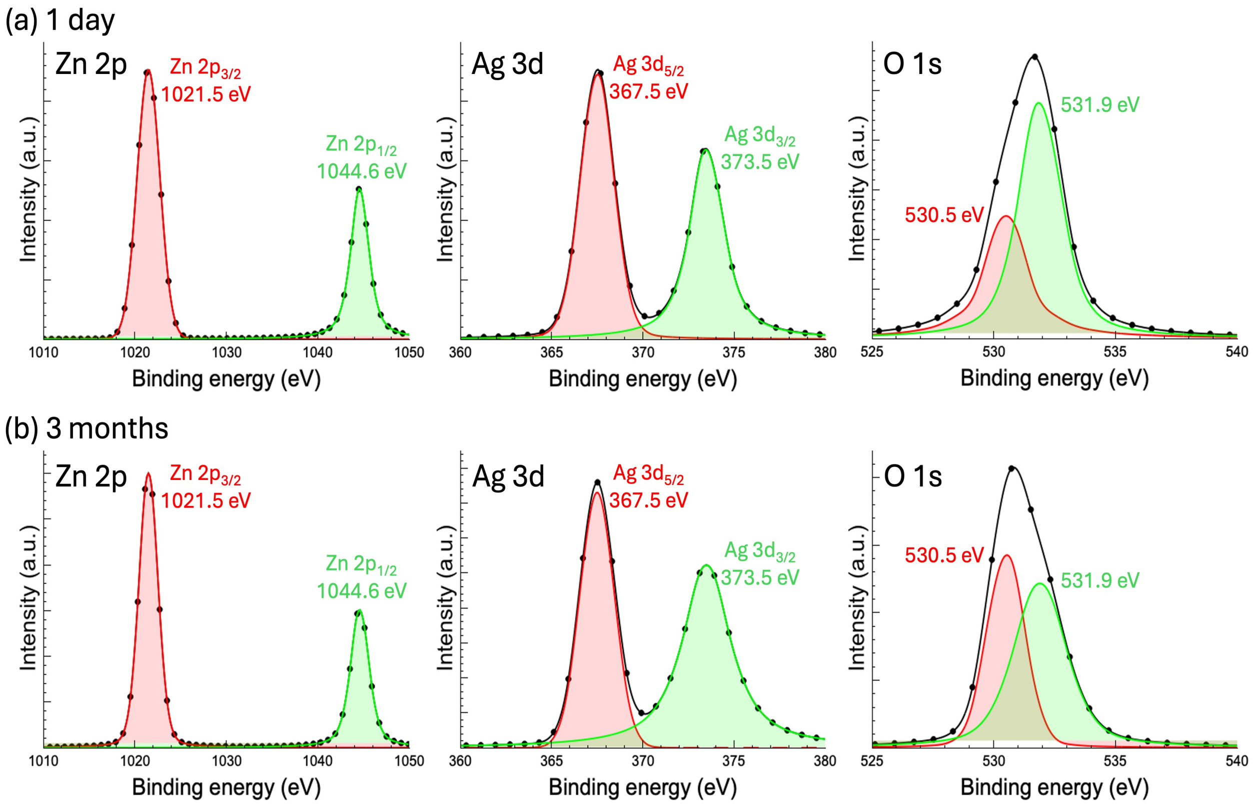

Figure S2. XPS analysis of the bonding states of Zn, Ag, and O elements on the surface of AgNP-decorated ZnO nanograss substrates. (a) XPS spectra of the sample stored in a dark environment for one day. (b) XPS spectra of the sample stored for three months. The solid points on the XPS spectra represent the measurement data points, and the lines connecting the points are spline fits.

Note S3. Optimizing Ag Thickness on ZnO Nanograss for SERS Enhancement

We optimized the Ag deposition thickness on ZnO nanograss substrates to enhance SERS performance. Non-patterned ZnO nanograss substrates were coated with 10 nm, 30 nm, and 50 nm of Ag, and the SERS signals of MG molecules at a concentration of 10^{-8} M were measured, as shown in Figure S3.

With a 10 nm Ag deposition, the coverage on the ZnO surface was insufficient, resulting in a low density of hot spots and weaker Raman signals. Increasing the thickness to 50 nm led to excessive Ag accumulation, causing nanoparticle aggregation and partial formation of a continuous metallic film. This reduced the number of effective hot spots and weakened the SERS intensity. In contrast, the 30 nm Ag layer achieved an optimal balance, promoting the formation of a high density of well-dispersed hot spots. These hot spots, combined with the high surface area of the ZnO nanograss structure, synergistically enhanced the SERS effect. The results showed that the substrate with a 30 nm Ag layer produced the strongest Raman signal, indicating the most effective SERS enhancement among the three configurations.

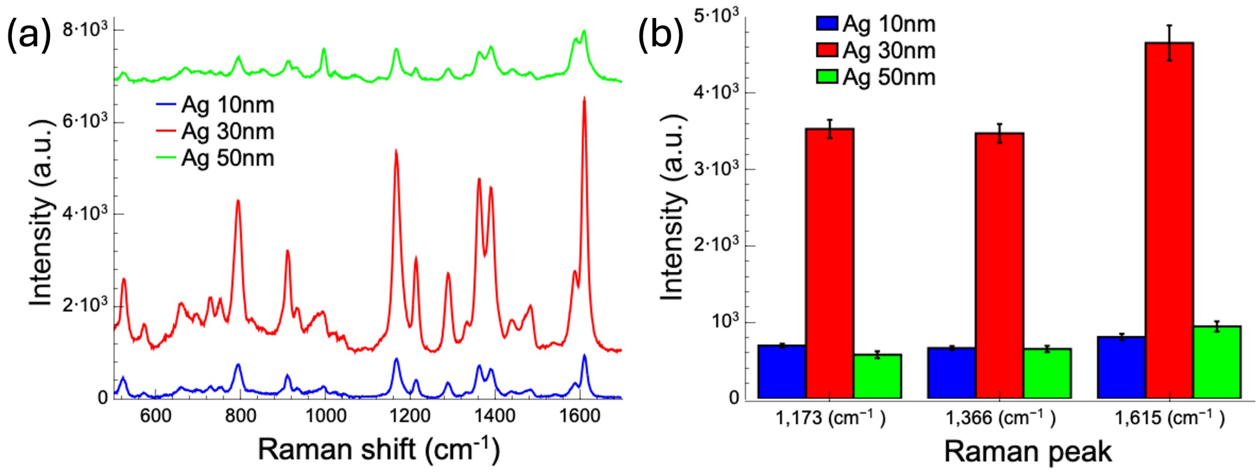

Figure S3. (a) SERS spectra of MG molecules (10^{-8} M) on ZnO nanograss substrates with different Ag deposition thicknesses (10 nm, 30 nm, and 50 nm). Spectra represent the average intensity from five measurement locations along the coffee ring. (b) The intensity of the Raman peaks is compared to evaluate the effect of Ag thickness on SERS performance.

Note S4. Comparison of SERS Intensity on AgNP-Decorated Substrates with Various Patterns

We compared three types of periodic nanostructures on ZnO nanograss substrates, including 1D grating and 2D dot and hole arrays, all with a period of 1000 nm and a line width of 500 nm. These were compared to a non-patterned ZnO nanograss substrate. The SERS signal intensity of MG molecules at a concentration of 10^{-8} M was measured to assess the impact of different patterns on SERS enhancement, as shown in Figure S4.

The results demonstrated that all periodic patterns significantly enhanced the Raman signal compared to the non-patterned substrate due to the light coupling and trapping mechanisms that improve light utilization and Raman scattering. Among the various patterns, the 1D grating structure exhibited the most significant enhancement, with a notably higher intensity than both the dot and hole array structures.

The enhanced SERS performance of the grating structure can be attributed to several contributing factors. The periodic, linear arrangement of the grating enables more effective coupling between the structural geometry and localized surface plasmon resonance (LSPR) modes, thereby optimizing optical resonance and generating stronger localized electromagnetic fields. The LSPR effect is likely due to the ZnO substrate being decorated with discrete AgNPs rather than continuous Ag films, which facilitates the excitation of surface plasmon polaritons. The resulting electromagnetic fields increase the density of plasmonic hot spots, leading to stronger Raman signal amplification.

Moreover, the directional nature of the grating structure allows for more efficient light coupling, in contrast to the isotropic scattering typically observed in dot or hole array configurations. This directional coupling improves light confinement and enhances field localization near the nanostructure surface. In addition, the grating geometry contributes to light trapping through pronounced diffraction effects, further reinforcing the intensity of the SERS signal.

Collectively, these optical and plasmonic effects suggest that the grating structure plays a critical role in concentrating light and enhancing plasmonic interactions, resulting in the most significant SERS signal improvement observed in this study.

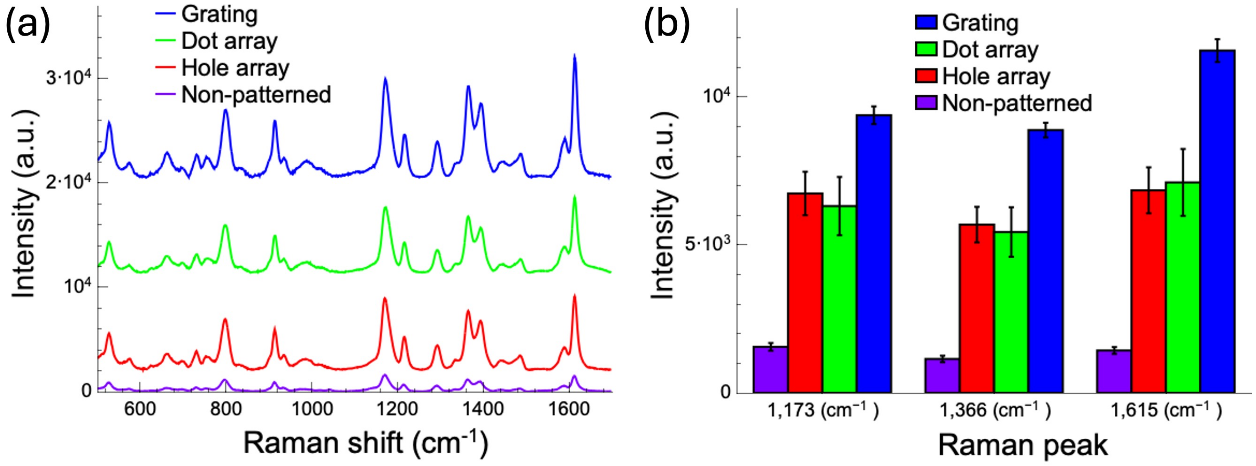

Figure S4. (a) SERS spectra of MG molecules (10^{-8} M) on ZnO nanograss substrates with non-patterned and periodic nanostructures, including 1D grating and 2D dot and hole arrays, all with a period of 1000 nm and a line width of 500 nm. The SERS spectra were averaged from five measurement locations along the coffee ring for each sample. (b) Comparison of the Raman peak intensities to assess the influence of different patterns on SERS enhancement.

References

(1) Samriti; Kumar, P.; Kuznetsov, A. Y.; Swart, H. C.; Prakash, J. Sensitive, Stable, and Recyclable ZnO/Ag Nanohybrid Substrates for Surface-Enhanced Raman Scattering Metrology. *ACS Materials Au* **2024**, *4*, 413-423.
(2) Li, Z.; Zhu, K.; Zhao, Q.; Meng, A. The enhanced SERS effect of Ag/ZnO nanoparticles through surface hydrophobic modification. *Applied Surface Science* **2016**, *377*, 23-29.
(3) Koleva, M. E.; Nedyalkov, N. N.; Nikov, R.; Nikov, R.; Atanasova, G.; Karashanova, D.; Nuzhdin, V. I.; Valeev, V. F.; Rogov, A. M.; Stepanov, A. L. Fabrication of Ag/ZnO nanostructures for SERS applications. *Applied Surface Science* **2020**, *508*, 145227.
(4) Zhu, X.; Wang, J.; Yang, D.; Liu, J.; He, L.; Tang, M.; Feng, W.; Wu, X. Fabrication, characterization and high photocatalytic activity of Ag-ZnO heterojunctions under UV-visible light. *RSC Adv* **2021**, *11*, 27257-27266.
(5) Feng, X.; Feng, L.; Jin, M.; Zhai, J.; Jiang, L.; Zhu, D. Reversible Super-hydrophobicity to Super-hydrophilicity Transition of Aligned ZnO Nanorod Films. *Journal of the American Chemical Society* **2004**, *126*, 62-63.
(6) Li, J.; Sun, Q.; Han, S.; Wang, J.; Wang, Z.; Jin, C. Reversibly light-switchable wettability between superhydrophobicity and superhydrophilicity of hybrid ZnO/bamboo surfaces via alternation of UV irradiation and dark storage. *Progress in Organic Coatings* **2015**, *87*, 155-160.
(7) Mardosaitė, R.; Jurkevičiūtė, A.; Račkauskas, S. Superhydrophobic ZnO Nanowires: Wettability Mechanisms and Functional Applications. *Crystal Growth & Design* **2021**, *21*, 4765-4779.
(8) Liu, H.; Feng, L.; Zhai, J.; Jiang, L.; Zhu, D. Reversible wettability of a chemical vapor deposition prepared ZnO film between superhydrophobicity and superhydrophilicity. *Langmuir* **2004**, *20*, 5659-5661.
